# Thyroid and Lipid Status in Guide Dogs During Training: Effects of Dietary Protein and Fat Content

**DOI:** 10.3390/ani9090597

**Published:** 2019-08-23

**Authors:** Biagina Chiofalo, Esterina Fazio, Salvatore Cucinotta, Cristina Cravana

**Affiliations:** Department of Veterinary Sciences, University of Messina, Polo Universitario Annunziata, 98168 Messina, Italy

**Keywords:** guide dog, dietary protein/fat ratio, training, thyroid hormones, lipid panel

## Abstract

**Simple Summary:**

Nutrition is one of the main causes of thyroid response and energetic metabolism. Presently, there is a lack of information on the physiological effect of moderate activities in canines, particularly in guide dogs. Aim was to compare the effect of diet on thyroid and lipid status in guide dogs, during a 12-weeks training, fed two commercial diets, one, HPF, characterized by low-carbohydrate/high-protein/high-fat (29:39:19% as fed) and the other, LPF, characterized by high-carbohydrate/low-protein/low-fat (50:24:12% as fed) content. Our hypothesis was that the intake of a diet rich in fat and protein would have given a better response than the carbohydrate-rich diet for thyroid and lipid homeostasis to cope with the increased energy demands of dogs subjected to the training period. Results evidenced that the consumption of a diet rich in fat and protein appears the nutritional plan most suitable to support moderate exercise for guide dogs during the training work; this diet supports maintenance of body weight, Body Condition Score (BCS), and serum baseline thyroid and lipid profiles, offering potential improvements in dogs’ performances. However, the chronic ingestion of diets that are extreme in their composition of either fat or carbohydrate should be always approached with caution.

**Abstract:**

Nutrition plays a leading role that most influences thyroid response and energetic metabolism. Aim was to compare the effect of diet on thyroid and lipid status in guide dogs during a 12-weeks training period. Eight Labrador Retrievers were divided into two groups homogeneous for sex, age, body weight, and Body Condition Score (BCS) and fed two commercial diets one, HPF, characterized by low-carbohydrate/high-protein/high-fat (29%:39%:19% as-fed) and the other, LPF, by high-carbohydrate/low-protein/low-fat (50%:24%:12% as-fed) content. The serum thriiodothyronine (T_3_), thyroxine (T_4_), cholesterol (CHOL), triglycerides (TAGs) and non-esterified fatty acids (NEFA) were determined at Day 0, 28, 56, and 84, before the daily training. Statistical model included the effects of Diet (HPF vs. LPF) and Time (Day 0 to Day 84), and their interaction. In the HPF group, Diet significantly (*p* < 0.01) increased T_4_, CHOL, and TAGs and decreased NEFA. In both groups, Time significantly (*p* < 0.05) increased T_4_ and TAGs, CHOL at Day 28, and NEFA at Day 56. The interaction did not influence serum hormones and lipid pattern. The adjustments in thyroid and lipid responses to moderate exercise in HPF group were driven mainly by the nutrient composition of the diet in relation to the involvement of metabolic homeostasis.

## 1. Introduction

Thyroid hormones are known to play a pivotal role in growth regulation, cognitive issues, cellular function and metabolic implication [1,2]. It was shown that the hypothalamic-pituitary-thyroid (HPT) axis activity decreases in response to food restriction, which is frequently interpreted to be an energy-saving mechanism [3]. It has also been speculated that energy signaling, like obesity and energy restriction, alters thyroid homeostasis in dogs, with significant higher T_3_ and T_4_ concentrations in obese dogs than lean dogs [4]. Moreover, the dietary protein quality and quantity content could change HPT axis activity; in particular, the long-term low-protein diet affects the thyroid axis activity, with the effect similar to that caused by starvation [5]. Some studies have shown a correlation between T_3_ concentrations and resting metabolic requirements [6,7]; nevertheless, no correlation between body weight and serum T_3_ concentrations was observed [4]. Moreover, serum concentrations of adiponectin were significantly and negatively associated with T_4_ concentrations, and positively correlated with cholesterol [8]. It is interesting to note that there is also an evidence that thyroid hormones and lipoprotein alterations may have a role in susceptibility of dogs to infectious diseases [9]. Thus, thyroid hormones in dogs may be involved in the regulation of fatty acid delta-6-desaturase activity [10]. The background of the thyroid signal transduction [11] and lipid metabolism in dogs exhibits some unique characteristics compared to other species, and hyperlipidemia is common in dogs [12]. 

The primary plasma changes, that are needed to support long-lasting activity, are related to lipid metabolism and, for this reason, the lipid pattern is frequently assayed during clinical evaluation [13]. Fatty acids are an important source of energy for skeletal contraction, particularly during exercise of mild-moderate intensity, prolonged duration and in the fasting state [14]. Plasma free fatty acids (FFAs) transported from remote adipose tissue stores and triglycerides (TAGs) contained within skeletal muscle fibers are the major sources of these fatty acids. The relative contribution of each source is dependent on the mode, intensity and duration of exercise and on training status. 

Cholesterol arrives in the small intestine from both the diet and bile. The liver –not the diet– is therefore the primary source of cholesterol available for intestinal absorption, a point that is often underappreciated [15]. 

The relative contribution of fat and carbohydrate changes according to intensity and duration of exercise, the physical training state and the environmental conditions in which the animal is working [16]. With light prolonged exercise, there is a progressively greater use of fat until it can contribute up to 80% of the caloric expenditure. Consumption of a diet rich in fat and protein produces a shift toward a greater use of fat, with a concomitant reduction of both the intensity and duration of effort that can be sustained. Conversely, ingestion of a carbohydrate-rich diet increases the percentage of carbohydrate used and increases endurance [17]. 

To date, canine athlete physiology studies have primarily focused on endurance sled dog racing and high intensity short duration Greyhound racing. There is a lack of information on the physiological and biochemical changes of low intensity endurance activities in canines, particularly in the guide dog [18]; this information is important in the determination of fitness level, detection of exercise-induced injury and improvement of success, with the development of more specific training programs [19]. 

Our hypothesis was that the intake of a diet rich in fat and protein would have given a better response than the carbohydrate-rich diet for thyroid and lipid homeostasis to cope with the increased energy demands of dogs subjected to the training protocol.

The aim of this study was to compare the dietary effect of two different concentrations of protein/fat/carbohydrates ratio on total iodothyronines and lipid panel in guide dogs for the blinds (GDB) during a 12-weeks training period.

## 2. Materials and Methods

Operative procedures and animal care were carried out in compliance with guidelines of Good Clinical Practices [20] and European regulation [21]. On the basis of the Italian regulation on animal experimentation and ethics [22], the research received the institutional approval by the Ethical Animal Care and Use Committee of the Department of Veterinary Science, of the University of Messina on 19 October 2016, Codex 006/2016 bis.

### 2.1. Animals and Diets

The study was carried out on eight neutered adult Labrador Retriever dogs, clinically health, housed at the Regional Centre Helen Keller (www.centrohelenkeller.it)of the Italian Blind and Guide Dog School Union, in Messina (Italy), during the training work to guide service for the blind. The Centre is unique in Italy and it is a member of the International Guide Dog Federation (IGDF), and as such accredited to the highest international standards.

The trial was carried out on dogs without a history of diabetes or hypo- or hyperthyroidism. Dogs admitted to the study were divided into two groups homogeneous for sex (2 male, 2 females), age (HPF: 18.3 months; LPF: 17.5 months), initial BW-body weight (HPF: 26.9 kg; LPF: 26.5 kg) and Body Condition Score-BCS (HPF: 4.33; LPF: 4.5, score 1–9). The first group, called HPF group, received a “performance” diet, characterized by low-carbohydrate/high-protein and fat diet (29%:39%:19% as-fed), whereas, the second group, called LPF group, received a “normal maintenance” diet characterized by high-carbohydrate/ low-protein and fat diet (50%:24%:12% as-fed).

Dogs were individually housed in pens of six square meters, adjacent to a large outdoor space where they could access during rest, and food was administered two times a day, in an individual bowl. 

The trial was preceded by 7 days of adaptation period to the experimental diets; anonymous was a normal maintenance diet usually used in the Centre along training work. The adaptation diet was constituted by the mixture of the feeds; the HPF group received a mixture of anonymous with “performance diet” and the LPF group a mixture of anonymous and “normal maintenance diet”; during the 7 days, the anonymous was progressively replaced by the experimental diets. The quantity of administered diet was the same previously adopted by the breeder. 

During the trial, the Company sent three lots of feed. Each lot was separately sampled and analyzed, as described by Chiofalo et al. [23]. 

Both the experimental diets of the Farmina Pet Foods line contained lamb meal as main protein source and, from a qualitative point of view, the same ingredients, analytical compounds, nutritional additives and antioxidants (tocopherol-rich extracts of natural origin). The information on the chemical composition of “performance” and “normal maintenance” diets is reported in the Table 1. 

The amount of feed daily administered to each dog was calculated on the ratio between the calculated metabolizable energy requirements, as proposed by Hand et al. [24], for dogs that perform work, characterized by a moderate duration and frequency and the caloric density of metabolizable energy (ME) reported in the label [25], of each diet (HPF and LPF).

### 2.2. Conditioning Protocol

All dogs were conditioned for training program activities one month prior to the dietary study starts. All dogs were between 1 and 2 years of age and had been conditioned for the training program to be guide dogs in the area described below (see Section 2.3) and they fed a typical maintenance ration (see Section 2.1). Conditioning and training protocols remained the same for all dogs during the dietary trials each of which lasted 12-weeks; thus, each dog served as its own control during the dietary trial. Moreover, the dogs were accustomed to the blood collection since, before the beginning of the trial, hematological and biochemical analyses were carried out on each subject to evaluate their health status. Moreover, before the beginning of the trial, in order to assess the clinical status, all dogs were also submitted to a physical examination [26].

### 2.3. Training Program

The method of participant recruitment is described by Lloyd et al. [27]. 

The training consisted of a various phases program in which the dog gradually learned more guide work. This included leading a person in a straight line, stopping at any change in ground elevation as well as overhead obstacles and obstacle avoidance. Feed rewards were used in the guide dogs for the blind training program as a powerful motivation and reinforcement tool for learning and maintaining desired behavior. During each training session (at Day 0, day 28; Day 56 and Day 84), dogs were introduced to specific guide-work behaviors:

- Stopping at streets, regardless of the type of curb or wheelchair ramp;
- Clearing the space around the handler on the right and left sides as well as above the dog’s head;
- Crossing streets on a line that efficiently reaches the up curb on the other side;
- Maintaining consistent pace and drive with the verbal cue “forward”;
- Responding to the various uses of the ‘hop-up’ verbal cue e resuming or increasing pace; moving closer to a stopping point; or re-focusing;
- Stopping and standing calmly after the verbal cue “halt”;
- Leading the handler in a 90_turn to the right and picking up the new travel line on “right”;
- Leading the handler in a 90_turn to the left and picking up the new travel line on “left”.

The guide dogs for the blind were trained 3 times a week. Each training session lasted approximately 60 min [23].

### 2.4. Physical Examination 

To evaluate the performance of the studied dogs, from the Day 0 (start of the administration of the new food) to the Day 84, all dogs weekly underwent to physical examination [26] including: level of consciousness; posture and gait; hydration status; rectal temperature (°C); pulse rate; respiratory rate and breath character; perfusion indicators. 

At the same time, on each animal, the BW and BCS were evaluated.

The determination of BW was measured on fasted animals, in the morning at 8:00 am, by using a digital scale.

BCS was evaluated by assigning a rating scale that ranged from 1 (too thin) to 9 (too heavy) using the table proposed by Nestle Purina [28,29,30].

### 2.5. Measurements of Hormonal and Lipid Patterns

In order to evaluate hormonal and lipid patterns in the fasting dogs, blood samples were monthly withdrawn, at Day 0, Day 28, Day 56, and Day 84 before the exercise (8:00 am).

Before the trial, the dogs were accustomed for the blood collection procedure (see paragraph *Conditioning protocol*). All samples were collected by the same operator into evacuated tubes (Venoject, Terumo^®,^ Shibuya, Tokyo, Japan) and were immediately refrigerated at 4 °C after collection; the samples were subsequently (within 1 h) centrifuged for 15 min at 1500× *g* and collected and stored at −20 °C until their analyses. Serum total iodothyronine concentrations were analyzed in duplicate using commercial immunoenzymatic assays (RADIM, Rome, Italy). The method is based on a competitive immunoenzymatic assay and the reagents were prepared as described in the manufacturer’s protocol. Total iodothyronines (T_3_ and T_4_) in the sample competed with T_3_ and T_4_ conjugated with horseradish peroxidase (conjugate) for binding to specific antibody sites of anti-T_3_ and anti-T_4_ coated on the wells. At the end of the incubation, all unbound material was removed by aspiration and washing. The enzyme activity which was bound to the solid phase would be inversely proportional to the concentration of T_3_ and T_4_ in calibrators and samples, and this was evidenced by incubating the wells with a chromogen solution (tetramethylbenzidine) in substrate buffer. Colorimetric readings were taken using a spectrophotometer at 450 nm (Sirio S, Radim/Seac Co., Rome, Florence, Italy). The sensitivities of the assays were as follows: 0 to12.3 nmol/L for T_3_, and 0 to 512 nmol/L for T_4_. The lower detection limits for T_3_ and T_4_ were 0.15 nmol/L and 12.8 nmol/L, respectively. The intraassay and interassay variance coefficients were 5.5% and 6.1% for T_3_ and 4.9% and 8.4% for T_4_, respectively.

Serum was analyzed for triglycerides (TAGs) using the enzymatic colorimetric method (GPO-PAP, glycerol-3-phosphate oxidase-p-aminophenazone) of McGowan et al. [31], for cholesterol (CHOL) using a modified Abell-Kendall/Levey-Brodie method [32] and for not esterified fatty acids (NEFA) by a coupled enzymatic reaction system (ACS-ACOD Method). First, Acyl CoA Synthetase (ACS) catalyzes fatty acid acylation of coenzyme A. Next, the acyl-CoA product is oxidized by Acyl CoA Oxidase (ACOD), producing hydrogen peroxide which reacts with the kit’s Colorimetric Probe. The colorimetric reading was taken using a spectrophotometer at 570 nm.

### 2.6. Statistical Analyses

To account for the study design, a mixed model analysis of variance [33] with the fixed effects of Time (Day 0, Day 28; Day 56 and Day 84) and Diet (HPF vs. LPF) was applied. The interaction (Diet × Time) was forced into every model. Random effects in the model were individual dog. Residuals were examined for normality; in each case residuals were normally distributed. Least Squares Means (LSM) and standard error of the mean (SEM) were calculated. The comparison between LSM were performed using the Tukey test. Differences were considered significant for *p* < 0.05. 

## 3. Results

The effect of environmental temperature is unlikely to play a significant role in this population’s energy requirement. It is known that the temperatures outside of the thermoneutral zone of 20 to 30 °C will increase the energy requirements by 1 to 5 kcal • BW^0.75^ per °C per day when above or below this zone [25]. During the 3 months of the study (1 March to 24 May), the dogs spent their time in thermoneutral zone (23 ± 2 °C); considering the kennel’s geographic location, it was unlikely that the temperatures at night dropped below the thermoneutral zone. 

### 3.1. Physical Examination and Body Weight

During the trial, the dogs presented adequate hydration status and rectal temperature within the physiological ranges (38.4 °C ± 0.32). The evaluation of pulse at femoral artery showed physiological characteristics about strength and quality, and the pulse rate within reference ranges for dogs (92 bpm ± 14) [26]. The mean of respiratory rate, determined visually or by auscultation as count either inspirations or expirations, was within the physiological ranges (18–29 ± 3) [26]. Mucous membrane color was pink, capillary refill time was less than 2 s. 

The results of the present study regarding dog’s performance were published by Chiofalo et al. [23]. Briefly, the diet influenced the animal performances (Table 2) in relation to their different protein, fat and carbohydrate contents, showing a significantly higher BW in the HPF group than those of the LPF group, as well as a significantly higher BCS in the HPF group than those of the LPF group. 

The BW and the BCS of dogs were monitored weekly during the whole time of the 12-week feeding period. As observed by Chiofalo et al. [23], no significant differences of BW in the dogs of the HPF group from Day 0 (26.9 kg) to Day 84 (25.40 kg) were observed, whereas, the BW in the dogs of the LPF group was affected by the time showing a significant (*p* < 0.05) decrease from the beginning to the end of the trial (Day 0: 26.5 kg; Day 84: 23.44 kg). The interaction Diet × Time showed no significant differences (*p* = 0.270). This could be due to the high variability of the BW in each group during the trial. The trend of the BCS showed no significant differences in relation to the Time (*p* = 0.997) and to the interaction Diet × Time (*p* = 0.991) for the whole trial period. 

### 3.2. Hormonal Response

As regards the trend of iodothyronine concentrations in relation to the diet (Table 3), after the 12-weeks diet intervention, T_3_ concentration was significantly not influenced whereas, T_4_ concentration showed significant higher mean level in HPF group than that observed in LPF group.

In relation to the variable Time (Table 4), the T_3_ response was not significantly influenced during the trial, whereas T_4_ concentration was significantly influenced, showing significant lower values at the Day 28 than those observed at Day 0, 56, and 84. 

The interaction Diet × Time showed no significant differences for T_3_ as well as for T_4_ concentrations.

### 3.3. Lipid Pattern

As regards the trend of lipid pattern in relation to the Diet (Table 3), after the 12-weeks diet intervention, CHOL and TAGs concentrations showed significant higher mean levels, whereas NEFA levels showed significant lower values in HPF group than those observed in LPF group.

CHOL, TAGs, and NEFA levels were significantly influenced by the Time (Table 4), showing in both groups the highest values at the Day 28 for CHOL and at Day 0 and 28 for TAGs and at Day 56 for NEFA. 

The interaction Diet × Time showed no significant differences for CHOL, TAGs, as well as for NEFA concentrations.

## 4. Discussion

The primary objective of this study was to examine how normal-weight Labrador dogs respond to HPF and LPF diets during a 12-week GDB training programs and to examine the relationships with the potential changes in circulating THs and lipid panel concentrations. It is reasonable to assume that the single components of diet may induce the metabolic changes, according to workload and performance quality. On these bases, the shift of energy metabolism in a catabolic or anabolic direction during training programs and exercise is characterized by a wide range of metabolic hormones changes, such as total THs, according to lipid parameters. Our hypothesis was that the HPF diet would have given a better answer than the LPF diet for thyroid homeostasis to cope with the increased energy demands of dogs subjected to the training protocol.

Guide dogs for the blind have a great social impact because of their invaluable aid in providing independent mobility to people with visual impairment; their service comes at high cost (approximately 25,000 euros) due to the large amount of resources, housing, husbandry and training, required to train such animals [34]. Furthermore, success rates ranged between 50% and 56% for dogs in training [35] contribute to large production costs. Although the most important skills to train in these dogs are obedience, they also must have an appropriate nutritional plan, in order to support physical fitness. Moreover, also the stress derived from the changes of life style (work and kennels condition) may negatively affect food intake and live weight [24], causing metabolic disorders and some significant modifications in laboratory parameters.

For guide dogs, a normal maintenance diet (crude protein = 20–23%; crude fat = 10%–12%) does not meet the requirements during their training work and the use of large amounts of feed is not recommended. The consumption of the “performance” diet, characterized by low-carbohydrate/ high-protein and fat diet (29%:39%:19% as-fed), seems to be more appropriated for light prolonged exercise than the ingestion of a normal maintenance diet rich in carbohydrates [17], limiting the weight loss in the HPF group, as observed for the dogs of the LPF group (−18%). Nevertheless, all the animals during the trial lost weight; this could be due to the training work for the service guide for the blind. Weight loss is normal in guide dogs during the training, according to the exercise and the life in kennel [23]. Moreover, considering then the Labrador retrievers may be genetically predisposed to obesity and consequently to the osteoarticular diseases [36], and considering the important role of GDB, they always maintain moderate body weight during the training program. 

Major depots of fat accumulation are present under the skin (as subcutaneous fat) and they can be readily observed and evaluated in dogs by using a BCS scale as indicator of the fat mass. If dietary energy intake is less than energy need, fat mass and BCS decrease. Conversely, if intake exceeds requirements, fat mass and BCS increase. This could explain our observation regarding a better BCS mean value of HPF group that that observed in LPF group. On the whole, all the animals showed a BCS within the ideal range (score 4 and 5). As reported by Hand et al. [24], a BCS of 2.5–3.5 (on a scale 1−5) is normal for more pets and for many canine athletes; the same authors observed that a much leaner body composition is desirable for some canine athletes. Even small excesses of body fat may represent an unnecessary handicap for working dogs. 

In the present study, the circulating T_3_ and T_4_, CHOL, TAGs, and NEFA concentrations are reported for the first time in clinically healthy Labrador Retriever guide dogs during training. The comparison of hormonal data with published ranges for dogs revealed that T_3_ and T_4_ concentrations were in agreement with physiological wide ranges reported in literature [37,38]. The results of the lipid pattern ranged within the reference values [39] in agility dogs undergo during exercise [19] and in dogs during low intensity endurance activity [18]. 

Korhonen [40] monitored the levels of THs, total lipids and urea of adult farmed raccoon dogs, and compared these parameters with BW and feed consumption during intense, maintenance and restricted fasting feeding. He observed a marked adjustment of thyroid hormones as the result of changes in subcutaneous fat reserves. This could explain the significant differences observed in our trial for T_4_ and BCS between HPF and LPF groups, according also to Eshratkhah et al. [41] that reported an influence of THs on lipid metabolism, through increasing lipolysis in adipose tissue and stimulating lipogenesis, by increasing the activities of some enzymes. In fact, the suitable function of thyroid gland is essential to metabolic regulations and for maintenance of the energy balance of body [42]. THs appear to contribute in the body energy balance, modulating the basal metabolic rate, primarily through actions in brain, heart kidney, liver, adipose tissue, and skeletal muscle [43]. The significant differences observed for T_4_ between the HPF and LPF groups confirm that thyroid hormones were influenced by altering feed intake, changes in subcutaneous fat reserves [40], such as by different diets in relation to the quantity and quality of nutrient contents [44]. 

The consistent tendency to decrease of the T_4_ concentrations in LPF diet group could be probably due to the continued weight loss showed by all dogs during the trial. This result confirmed previous studies related to a decrease in T_3_ and T_4_ concentrations in dogs undergoing a weight loss protocol [4]. It is well established that thyroid hormone status correlates with BW and energy expenditure [41]. This could be an energy-saving mechanism related to a down regulation of HPT axis activity in condition of caloric deprivation [3]. In human and rats, it’s been previously reported that during food or caloric restriction, total body energy expenditure can slow down with an adaptive decrease resulting in a fall in circulating THs [6,7]. Iodothyronines modulate the fat metabolism, and alterations in T_4_ may reflect increased lipolysis to offset reduced feed intake [2]. The significant decrease in T_4_ and no change in T_3_, in response to reducing energy intake, were unexpected. However, our data confirmed other studies that have examined the HPT axis response to starvation with a decrease in Thyroid Releasing Hormone, Thyroid Stimulating Hormone-β gene expression and circulating THs in rodents and human [3]; the decreased T_3_ may be primarily due to diminished thyroidal secretion of T_4_ or by increased Desiodinase (D_3_) activity in the liver, kidney, and muscles of starved rats [45].

A particular note should be done on the thyroid response to variable diet. Only at the last of the trial (Day 56 to Day 84), data showed a significant higher basal concentration of T_4_ in HPF group than the mean value of LPF group. The positive value trend might relate to a reduced catabolism resulting from decrease lipoprotein lipase activity [46], which determined a positive energetic balance of the animals fed the “performance” diet rich in fat and protein. 

These results could indicate that adjustments in thyroid function and related consistent increase of circulating T_4_ concentrations in HPF group, that were driven mainly by the nutrient composition of the diet in relation to the involvement of THs in the synthesis, mobilization, and degradation of lipids [47].

As TAGs are the most important type of fat in the diet and the body’s primary for stored energy, during prolonged exercise and when energy intake is insufficient, they are metabolized in FFAs determining an increase of NEFA in the blood which became a primary energy source for long-lasting exercise [48]. Plasma FFA oxidation is directly related to the rate of lipolysis in adipose tissue [14]. Their oxidation can contribute 50 to 60 per cent of the energy expenditure during a bout of low intensity exercise of long duration [49]. This could confirm the better BCS and the lower NEFA levels recorded in the HPF than LPF groups related to the more adequate energy content of the “performances” diet than that of the “normal maintenance” carbohydrate-rich diet. Moreover, the results of this study indicate that the diet induces significant changes in TAGs concentrations. Circulating TAGs could be a potential source of fatty acids for ß-oxidation in working muscle, especially in animals in the fed state [50]. The rise in TAGs concentration after exercise depends on the intensity of exercise and the activity of lipolysis, although FFA concentrations are considered to be a better indicator of lipid metabolism [51]. The “normal maintenance” diet, characterized by high-carbohydrate/low-protein and fat diet (50%:24%:12% as-fed), increased blood TAGs, as effect of lipolysis stimulation inducted from the high request of energy during the metabolic adaptations that occur in skeletal muscle and adipose tissue, and that facilitate a greater delivery and oxidation of fatty acids during exercise. Our results are in accordance with Askew [49] and Kaciuba-Uścilko et al. [52] that observed a markedly enhancement of FFA mobilization modulated by the thyroid hormones, in relation to a decreased feed intake.

Total CHOL concentration is routinely measured during health checks in small animal clinics [13]. The total serum concentration of CHOL has been recognized as a potential biomarker for various processes related to lipoproteins metabolism [15]. Fialkovičová et al. [53] reported that THs have catabolic effects on muscle and adipose tissue and regulate CHOL synthesis and degradation; they are essential for an appropriate degree of metabolic activity, including generation and release of energy. It is possible presume that the higher CHOL levels of the HPF than LPF groups, would be probably correlated to a greater intestinal absorption of medium and long chain fatty acids, which would be esterified in situ and introduced again as lipoproteins and chylomicrons into the blood, testifying to an improvement in the intestinal absorption of the nutrients of HPF group. 

Our data are not in accordance with Bruss’ observations [54] where serum CHOL level generally varies inversely with thyroid activity. However, there are some contradictory findings regarding the relation between serum THs, CHOL, and TAGs; the concentrations of THs were not correlated with CHOL levels in some other animals [41,55,56]. Although the role of thyroid hormones is well known in many species, there is little evidence describing the relationship between thyroid hormones status and serum profiles of CHOL, TAGs, and NEFA in dogs [57]. 

This could probably due to the daily rhythmicity of total lipids, total CHOL, phospholipids, and TAGs that occurs in some animals and that vanished when dogs were food-deprived, indicating that these rhythms are driven by the digestive process [58]. 

Finally, literature data [54,57] report that changes in concentrations of THs in some animal species are due to the effect of temperature and season. Our trial was carried out in spring and the dogs spent their time in thermoneutral zone therefore, we think the environmental temperature could not have influenced the energy requirements, the functional activity of the thyroid gland and the concentration of THs [57]. 

On the whole, the significant higher values of BW, BCS, TAGs, and CHOL, together with T_4_ concentrations, and the significant lower NEFA concentrations in the HPF group, testified a better physical fitness of the animals fed the “performance” diet [59].

## 5. Conclusions

Clinical biochemistry parameters are of major interest in canine sport medicine to assess health status and fitness level, as well to monitor the mental and physical stress imposed by exercise. Presently, there is a lack of information on the physiological effect of moderate activities in canines, particularly in the guide dog. 

Furthermore, for working dogs, the lifetime cost of feed, even if specially formulated, represents a trivial fraction of the monetary investment in training. It is worth noting that guide dogs for the blind are expensive to train, as well as being expensive in personal terms for all concerning if the post-qualification period is unsuccessful; thus, this research is intrinsically leading for the guide dog trade in several ways. The knowledge of metabolic changes is essential in order to design specific and individual training protocols, for an early diagnosis of poor performances, to assess the impact of different feeding or supplementation strategies and to minimize the risk of exercise-linked disease. Results evidenced that the consumption of a diet rich in fat and protein appears the nutritional plan most suitable to support moderate exercise for guide dogs during their training work, supports maintenance of BW, BCS, and serum baseline thyroid and lipid profiles, contrasting the mobilization of subcutaneous fat reserves, and offers potential improvements in challenging work situations. However, the chronic ingestion of diets that are extreme in their composition of either fat or carbohydrate should be always approached with caution. 

## Figures and Tables

**Table 1:** Chemical composition and metabolizable energy of the diets ^1^.

Diet ^2^	Anonymous ^3^	HPF ^4^		LPF ^4^	
Moisture, g/100g as-fed	9.0	5.42	0.48	6.12	0.57
CP, g/100g as-fed	26	39.24	0.84	24.40	0.32
Fat, g/100g as-fed	15.50	18.69	0.51	11.78	0.29
OM, g/100g as-fed	ND	86.83	0.27	86.50	1.20
TDF, g/100g as-fed	2.80	11.59	1.13	13.03	1.46
Ash, g/100g as-fed	4.9	7.91	0.23	7.51	0.55
ME ^3^, kcal/kg as-fed	3900	4330	-	3423	-

CP = Crude Protein; OM = Organic Matter; TDF = Total Dietary Fibre; ME = Metabolizable Energy. ND = Not Determined; ^1^ Values are means ± standard deviation; ^2^ Anonymous was the normal maintenance diet usually used in the Centre along training work; HPF was the “performance” diet with low-carbohydrate/high-protein/high-fat diet and LPF was the “normal maintenance” diet with high-carbohydrate/low-protein/low-fat diet administered during the trial; ^3^ Values reported on the label; ^4^ Values determined analytically.

**Table 2:** Effect of the diets on BW and BCS of the trial ^1^.

Items	Groups ^2^				*p*-Value ^3^
	HPF	SEM	LPF	SEM	
BW, kg	25.40 ^a^	0.21	23.44 ^b^	0.26	<0.001
BCS, score 1–9	4.64 ^a^	0.12	4.01 ^b^	0.15	0.003

BW = Body weight; BCS = Body Condition Score; ^1^ Values are means (LSM) ± standard error of the mean (SEM); ^2^ HPF = low-carbohydrate/ high-protein and fat diet; LPF = high-carbohydrate/low-protein and fat diet; ^3^ Probability values for the effects of Diet; ^a, b^ Within a row, means with different superscript letter were significantly different (*p* < 0.05).

**Table 3:** Effect of the diet on serum hormonal and lipid panel concentrations for the whole trial period ^1^.

Items	Groups ^2^				*p-*Value ^3^
	HPF	SEM	LPF	SEM	
T_3_ (nmol/L)	2.64	0.83	2.48	0.83	0.617
T_4_ (nmol/L)	33.19 ^a^	1.12	29.10 ^b^	1.17	0.003
CHOL (mg/dL)	175.82 ^a^	3.34	141.43 ^b^	4.98	0.001
TAGs (mg/dL)	53.98 ^a^	2.33	42.06 ^b^	2.44	0.006
NEFA (mg/dL)	0.50 ^b^	0.04	0.64 ^a^	0.04	<0.001

T_3_ = Thriiodothyronine; T_4_ = Thyroxine; CHOL= Cholesterol; TAGs= Tryglicerides; NEFA= Non Esterificated Fatty Acids; ^1^ Values are means (LSM) ± standard error of the mean (SEM); ^2^ HPF = low-carbohydrate/high-protein and fat diet; LPF = high-carbohydrate/low-protein and fat diet; ^3^ Probability values for the effects of Diet; ^a, b^ Within a row, means with different superscript letter were significantly different (*p* < 0.05).

**Table 4:** Profile of serum hormonal and lipid panel concentrations during the trial ^1^.

	Groups ^2^	Time ^3^				SEM	*p*-Value ^4^		
		0	28	56	84		Diet	Time	D × T
T_3_ (nmol/L)	HPF	2.58	2.69	2.65	2.65	0.39	0.617	0.963	0.872
	LPF	2.42	2.54	2.50	2.50	0.38			
T_4_ (nmol/L)	HPF	32.06 ^a^	28.69 ^b^	36.44 ^a,x^	34.30 ^a,x^	1.19	0.003	0.023	0.274
	LPF	33.91 ^a^	27.77 ^b^	29.23 ^b,y^	29.47 ^b,y^	1.16			
CHOL (mg/dL)	HPF	172.60 ^b^	182.40 ^a^	168.00 ^b,x^	180.60 ^a,x^	3.82	0.001	0.031	0.449
	LPF	161.50 ^b^	176.50 ^a^	131.50 ^by^	154.00 ^b,y^	4.54			
TAGs (mg/dL)	HPF	64.30 ^a^	60.30 ^a,x^	42.33 ^b^	48.00 ^b^	2.12	0.006	0.002	0.373
	LPF	54.00 ^a^	46.00 ^a,b,y^	42.00 ^b^	32.00 ^b^	2.34			
NEFA (mg/dL)	HPF	0.26 ^b^	0.54 ^b,y^	0.70 ^b^	0.53 ^b,y^	0.03	<0.001	<0.001	0.126
	LPF	0.36 ^b^	0.62 ^b,x^	0.82 ^b^	0.74 ^a,x^	0.03			

T_3_ = Thriiodothyronine; T_4_ = Thyroxine; CHOL= Cholesterol; TAGs= Tryglicerides; NEFA= non-esterified fatty acids. ^1^ Values are means (LSM) ± standard error of the mean (SEM); ^2^ HPF = low-carbohydrate/ high-protein and fat diet; LPF = high-carbohydrate/low-protein and fat diet; ^3^ Blood sampling at Day 0, Day 28, Day 56, and Day 84, before the exercise; ^4^ Probability values for the effects of Diet, Time, and Diet × Time; ^a,b^ Within row, means with different superscripts letter were significantly different (*p* < 0.05) due to time; ^x,y^ Within column, means with different superscript letter were significantly different (*p* < 0.05) due to diet.
